# Low-Voltage High-Frequency Lamb-Wave-Driven Micromotors

**DOI:** 10.3390/mi15060716

**Published:** 2024-05-29

**Authors:** Zhaoxun Wang, Wei Wei, Menglun Zhang, Xuexin Duan, Quanning Li, Xuejiao Chen, Qingrui Yang, Wei Pang

**Affiliations:** The State Key Laboratory of Precision Measuring Technology and Instruments, Tianjin University, Tianjin 300072, China; wangzhaoxun@tju.edu.cn (Z.W.); wei_wei@tju.edu.cn (W.W.); xduan@tju.edu.cn (X.D.); qn.li@tju.edu.cn (Q.L.); chenxuejiao@tju.edu.cn (X.C.); yangqingrui@tju.edu.cn (Q.Y.)

**Keywords:** high frequency, Lamb wave, micromotors

## Abstract

By leveraging the benefits of a high energy density, miniaturization and integration, acoustic-wave-driven micromotors have recently emerged as powerful tools for microfluidic actuation. In this study, a Lamb-wave-driven micromotor is proposed for the first time. This motor consists of a ring-shaped Lamb wave actuator array with a rotor and a fluid coupling layer in between. On a driving mechanism level, high-frequency Lamb waves of 380 MHz generate strong acoustic streaming effects over an extremely short distance; on a mechanical design level, each Lamb wave actuator incorporates a reflector on one side of the actuator, while an acoustic opening is incorporated on the other side to limit wave energy leakage; and on electrical design level, the electrodes placed on the two sides of the film enhance the capacitance in the vertical direction, which facilitates impedance matching within a smaller area. As a result, the Lamb-wave-driven solution features a much lower driving voltage and a smaller size compared with conventional surface acoustic-wave-driven solutions. For an improved motor performance, actuator array configurations, rotor sizes, and liquid coupling layer thicknesses are examined via simulations and experiments. The results show the micromotor with a rotor with a diameter of 5 mm can achieve a maximum angular velocity of 250 rpm with an input voltage of 6 V. The proposed micromotor is a new prototype for acoustic-wave-driven actuators and demonstrates potential for lab-on-a-chip applications.

## 1. Introduction

Compared with micromotors driven by electrostatic [1,2,3] or magnetic forces [4,5], acoustic-wave-driven motors [6,7] have attracted considerable attention owing to their high energy density, precision, and ease of integration [8]. Specifically, their miniaturization meets the microfluidic actuation needs of emerging small-scale applications such as microrobots, portable drug delivery systems, and lab-on-a-chip devices for biomedical applications [9,10]. In the widespread application of acoustic drive motors, surface acoustic wave (SAW) technology has become prevalent in their driving scheme, in which SAWs propagate along the piezoelectric substrate and couple into the liquid layer, inducing acoustic streaming that drives motor rotation [11]. For example, focused interdigital transducers (FIDTs) have been specifically fabricated to generate SAWs with a high density and beamwidth compression ratio. This motor achieve an ultrahigh rotational speed relative to its diameter [12]. Additionally, Zhang et al. [13] developed an innovative design utilizing a pair of FIDTs fixed on opposite slanted substrates, in which the Rayleigh angle of the leaky SAW was leveraged to reduce the threshold voltage and achieve a maximum angular velocity of 250 rpm.

**Figure 1:**
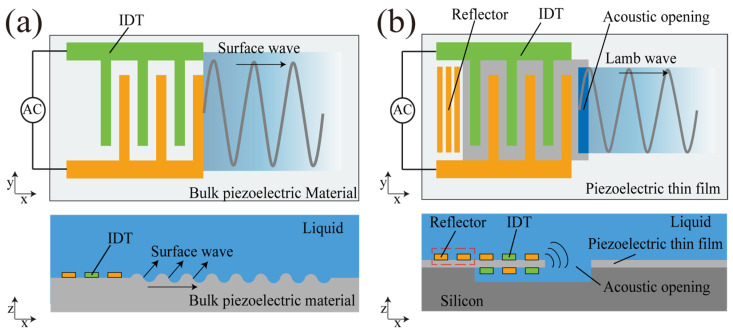
Comparison of the working principles of SAW and Lamb wave actuators. (**a**) Acoustic streaming working principle based on an SAW actuator. (**b**) Acoustic streaming working principle based on a Lamb wave actuator.

However, current SAW-driven motors suffer from a high input driving voltage and a large size. Figure 1a presents a schematic diagram of the acoustic streaming induced by an SAW device. The SAW is excited and coupled into the fluid through scattering, but a large portion of the energy leaks along the substrate, reducing its driving energy efficiency in the fluid. Thus, a high driving voltage of typically more than 20 Vpp is needed [13], which necessitates complex circuit systems and a heat sink to accommodate high-power devices. Furthermore, a specific coupling distance is required for acoustic streaming generation in SAW actuators; consequently, typical SAW-driven actuators are placed around the rotor. Therefore, the overall size of the motor is significantly larger than the size of the rotor, with typical dimensions of 15 mm × 60 mm [14,15].

Lamb-wave-driven motors perform better than SAW-driven motors in terms of size and efficiency (Figure 1b). Firstly, high-frequency Lamb waves generate strong acoustic streaming effects over an extremely short distance, leading to a high driving energy efficiency in the fluid. Secondly, Lamb wave actuators are vertically coupled to the rotor through the liquid layer, enabling a remarkable reduction in the micromotor size. In addition, Lamb waves excited in the actuator are partially transferred into the liquid through the solid—liquid interface at the outer edge of the electrode strip acoustic opening. The sound wave on the back side is reflected back to the acoustic opening through the reflector on the back side, enhancing the efficiency of energy use. Lamb wave actuators utilize both horizontal and vertical electrodes in creating static capacitance, which is a key difference from SAW actuators. SAW devices typically rely solely on horizontal interdigitated electrodes positioned over the substrate for their static capacitance needs. Consequently, to attain desirable impedance properties, SAW actuators often necessitate larger dimensions compared to their Lamb wave counterparts. High-frequency Lamb waves of 380 MHz, generated by an actuator array as small as 5 mm in diameter, drive the rotor to a rotation speed of 250 rpm with an input driving voltage of only 6 V. Compared with SAW-driven motors, Lamb-wave-driven motors feature a low-voltage drive and a reduced size.

## 2. Materials and Methods

**Figure 2:**
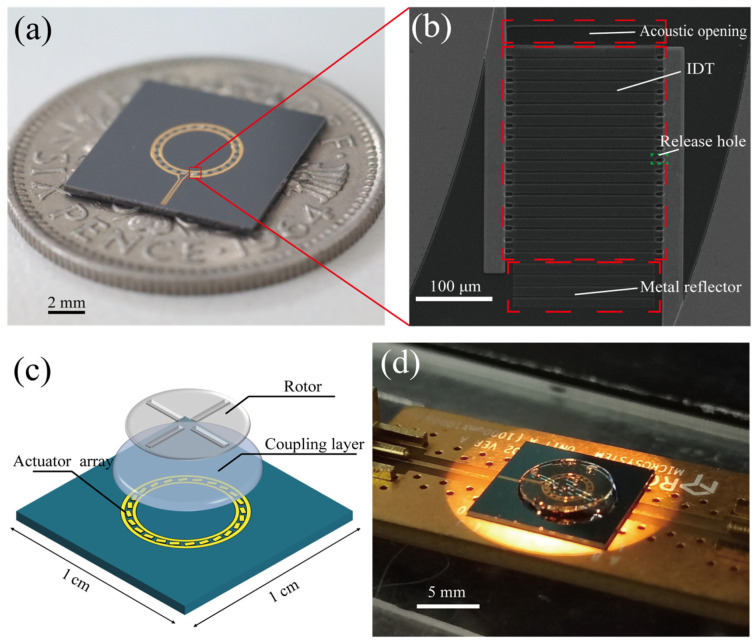
Lamb-wave-driven motor device. (**a**) Physical image of the Lamb wave actuator array on a coin. (**b**) SEM image of the Lamb wave actuator device used in the experimental array. (**c**) Schematic diagram of the Lamb-wave-driven micromotor. (**d**) Photo of the micromotor assembled on the connection board used in the experiment.

Figure 2a shows a schematic diagram of a device measuring 1 cm × 1 cm in size, in which twenty-four Lamb wave actuators are connected in parallel by a bus to form an array. The specific details of the device are shown in Figure 2b, and the specific fabrication process is shown in the Supplementary Materials. The IDT of each Lamb wave actuator contained 9 pairs of fingers, the pitch was 13 microns, and the device aperture was 200 μm. The reflector at the front of the device had an acoustic opening, whereas that at the back was a metal reflector. The entire motor structure was composed of Lamb wave actuators, a coupling layer and a rotor (Figure 2c). The rotor was made from an SU-8 photoresist with a thickness of 200 μm and then patterned via one-step photolithography. Figure 2d shows the experimental setup, where a high-frequency voltage generated by the signal generator is connected to the motor by a connection board.

The Lamb wave actuators were arranged according to certain angles and numbers of turns, which led to the azimuthal flows generated by each Lamb wave actuator overlapping into one central vortex. Consequently, the fluid was driven to move, producing shearing and pushing effects on the rotor. Droplets of approximately 50 μL in volume were applied in the central region of the Lamb wave actuators and then the rotor was carefully placed on the water droplets to maintain a balanced state. When the AC signal was turned on, the superposition of the unidirectional acoustic streams caused the rotor to rotate at a certain speed, thus producing an angular velocity, and the speed was measured via a high-speed camera.

## 3. Results

To analyze the factors influencing the rotational speed of the device, a finite element analysis (FEA) was performed based on the equations presented below, including the Navier—Stokes equation, which accounts for the viscosity of the fluid and the differential pressures within the fluid environment. The viscosity quantifies the resistance of the fluid to deformation. This equation is used to determine the fluid velocity field, where *v* represents the velocity, *p* denotes the pressure, and *η* is the viscosity of the fluid.

(1) ∇·v=0

(2) $$\rho \frac{\partial v}{\partial t}+\rho (v\cdot \nabla )v=-\nabla p+\mu \nabla^{2}v+\rho f$$

(3) $$SPL=10\log_{10}\frac{U^{2}}{U_{ref}^{2}}+{SPL}_{ref}=20\log_{10}\frac{p}{p_{0}}$$

where *SPL* represents the sound pressure level and *U* corresponds to the input power of the device. *U_ref_* is the reference input power. *SPL_ref_* represents the reference sound pressure level at the reference input power. *p*_0_ is the reference pressure in water and is set to 1 μPa. Through piezoelectric simulations, the *SPL_ref_* corresponding to a sound pressure of 10 Pa under a reference voltage of 6 V was established. This value served as the baseline for quickly determining the initial pressure of the device at various power levels.

(4) $$p(r,t)\sim \frac{e^{-(ik+\beta )\sqrt{x^{2}+y^{2}}}}{{\left(x^{2}+z^{2}\right)}^{\frac{1}{4}}}sin\left(\frac{\pi }{L}y\right)e^{i\omega t}$$

where *r* represents the three-dimensional spatial position relative to the center of the acoustic opening of the device, *β* is the fluid attenuation factor and *t* denotes time. By solving the Navier–Stokes equation, the fluid motion within the upper coupling layer can be determined. The force exerted on the rotor during this process can be described by the following equation:

(5) $$F=\int \mu \frac{\partial v}{\partial \theta }dr$$

where *F* is the resultant force on the rotor. For most cases, a faster flow rate will increase the flow speed.

**Figure 3:**
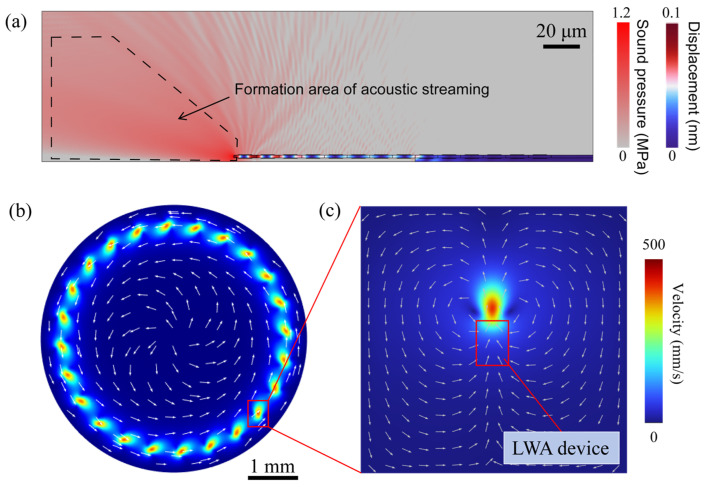
FEA analysis of a single Lamb wave actuator and Lamb wave actuator array. (**a**) The average value of single device vibration and sound pressure. (**b**) The overall flow field distribution of a Lamb wave actuator array. (**c**) Schematic diagram of flow field distribution for a single Lamb wave actuator.

The simulation results of individual devices and arrays are shown in Figure 3a, which illustrates the displacement generated by the device’s vibration and the acoustic pressure distribution created in the fluid. When a Lamb wave actuator operates in a liquid environment, an optimized grating reflector is employed to substitute the air boundaries of the LWR, thereby directing the acoustic energy of the Lamb waves from only one face of the resonator into the liquid. Figure 3b reveals an array comprising 24 Lamb wave actuators that collectively excite an acoustic fluid flow, forming a large annular fluid vortex. As depicted in Figure 3c, the most potent acoustic streaming effect is observed in close proximity to the resonator device, consistent with the attenuation of acoustic waves in the fluid domain and the finite extent of the volume force. Specific simulation details and settings can be found in the Supplementary Materials.

**Figure 4:**
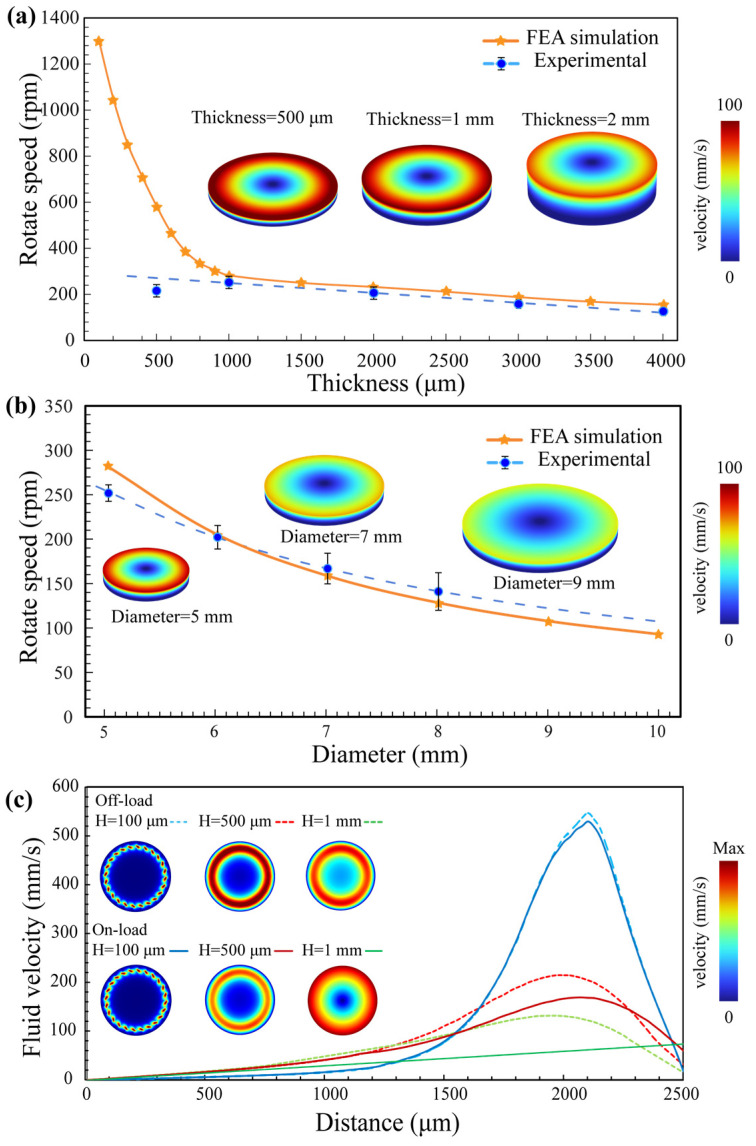
Speed analysis under a fixed actuator array size and structure. (**a**) Top rotational speed of the device as a function of the liquid coupling layer thickness. (**b**) Top rotational speed of the device as a function of the rotor diameter. (**c**) Simulated velocity profiles at various horizontal cross-sections from the center to the edge of the device when the coupling layer thickness is 1 mm. The selected velocity images correspond to the bottom (H = 100 μm), center (H = 500 μm), and top (H = 1 mm) of the coupling layer in both the loaded and unloaded rotor states. In all images, FEA was used to simulate the fluid velocity field, and a rainbow color scheme was applied to the images, with red representing the maximum values and blue representing the minimum values.

The optimal operating conditions with a maximized rotational speed were found by FEA results and experimental validation and specific rotational speed measurement can see Supplementary Materials. With the array diameter fixed at 5 mm, we investigated the effects of the coupling layer thickness and loading conditions on the device performance and statistically analyzed the results (Figure 4a). By increasing the coupling layer thickness from 100 μm to 1 mm, the motor speed sharply decreased with an increasing thickness. Specifically, a fluid layer thickness of 100 μm yielded a rotational speed of 1400 rpm. Simulations revealed that less fluid resulted in a faster fluid motion. However, in practical applications, the thickness of the liquid should not be less than 0.5 mm (approximately 25 μL) because of irregularities on the rotor surface. During the experiments, a thickness of 4 mm (approximately 200 μL) resulted in slower rotor rotation compared to a thinner layer. A significant amount of energy was dissipated to the rotor in the case of a thick coupling layer. Therefore, the coupling layer thickness is strongly related to the rotation speed, with the experimental optimal thickness being approximately 1 mm. Figure 4b shows the influence of different rotor diameters on the motor rotation speed. The experimental results were consistent with simulation predictions. A larger rotor diameter increased the load, which reduced the speed. The rotor needs to closely match the size of the actuator array to obtain the best rotation performance.

## 4. Discussion

Furthermore, as shown in Figure 4c, when the device was loaded, the linear velocity of the fluid at the top surface of the liquid coupling layer followed a linear distribution, in which the speed increased closer to the rotor periphery. In contrast, at the bottom and middle of the liquid, faster speeds occurred near the device. This nonuniform velocity distribution further limits increases in the rotor speed. Regardless of whether the device was loaded or unloaded, the maximum fluid velocity within the coupling layer reached 500 mm/s, which translated to rotational speeds exceeding 2000 rpm. These results have significant implications for micromotors. For the case of a 1 mm thick loaded coupling layer, the simulated maximum linear velocity at the top was 73 mm/s, corresponding to a rotational speed of 282 rpm, which closely matched the highest measured value of 252 rpm, thus validating our simulation.

**Figure 5:**
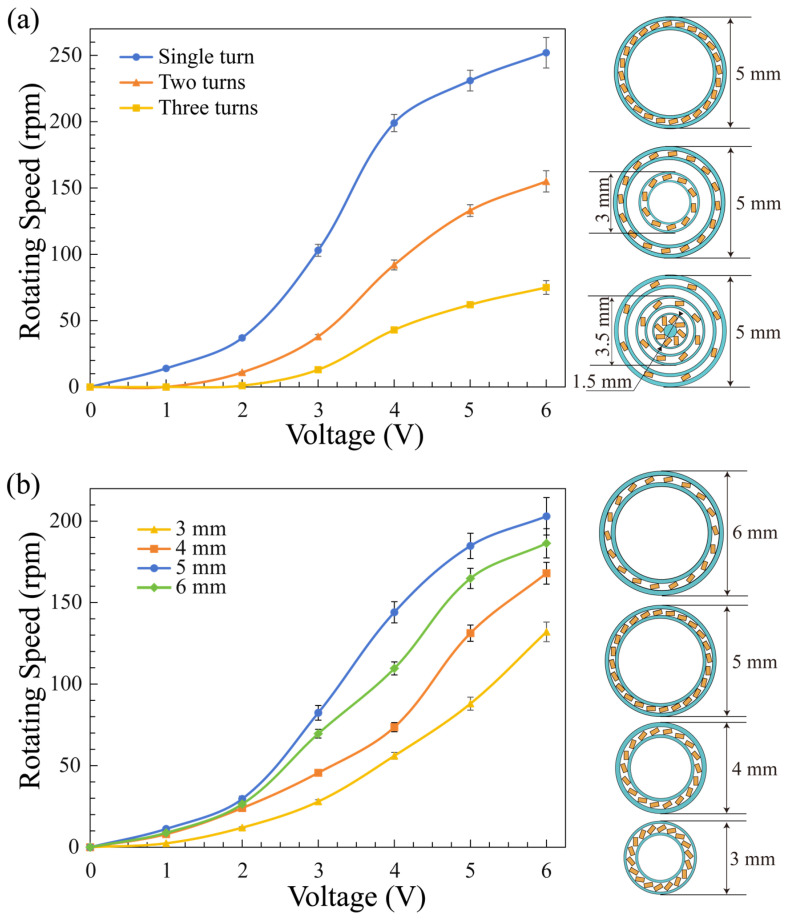
Influence of the actuator array design on the rotational speed. (**a**) Relationship between the rotation speed and input voltage when the number of Lamb wave actuators is the same but the array diameter differs. The diameters of the Lamb wave actuator array and rotor are both 5 mm. (**b**) Rotor angular velocity versus input voltage for different array diameters. The diameter of the rotor is fixed at 6 mm.

To study the influence of different Lamb wave actuator array structures on the rotor speed, we fixed the number of actuators and designed devices with various numbers of turns and Lamb wave actuator array diameters. For the two-turn type, the total number of Lamb wave actuators in the array was 24, with 10 actuators in the inner circle and 14 in the outer circle. For the three-turn type, there were eight actuators in each circle. As depicted in Figure 5a, the rotational speed significantly decreased with an increasing number of turns. With the total number of Lamb wave actuators fixed, an increase in the number of turns results in an excessively disperse distribution of Lamb wave actuators. This relationship is further illustrated by a comparison of two arrays with the same Lamb wave actuator arrangement but different diameters, with the 5 mm diameter actuator array showing the best performance (Figure 5b). For densely packed devices with 3 mm and 4 mm diameter arrays, although the angular velocity is fast when the rotor is unloaded, the relatively larger loading of the rotor on the actuator arrays results in a decrease in the speed due to insufficient torque. For 6 mm diameter arrays, the density of the resonator is too low to generate well-gathered acoustic streaming, which will also lead to a decrease in the driving ability of the motor. It is concluded that the density and torque of Lamb wave actuators within a ring array configuration are critical parameters, with higher densities and larger torques being conducive to achieving high rotational speeds.

To further enhance the Lamb-wave-driven micromotor performance in the future, the following improvements are suggested: (1) Reduce the thickness of the coupling layer. The utilization of lighter fluids, such as lightweight oils, can prevent vaporization and enable a reduction in the coupling layer thickness while preventing speed reductions due to motor stalling. (2) Improve the device distribution. A more uniform angular velocity can be achieved by optimizing the arrangement of the devices. (3) Use a low-density arrangement of low-power devices at the center and a high-density arrangement of high-power devices at the edges. By implementing these enhancements, the motor can achieve better usability.

## 5. Conclusions

In this study, we demonstrated an acoustic-wave-driven micromotor based on Lamb wave actuators. The Lamb wave actuators were designed with a novel structure in which the acoustic opening on one side was replaced with a reflector. The vertically constructed device consisted of a circular array of Lamb wave actuators, with only one main vortex in the center, a fluid coupling layer of 50 μL, and a rotor. The smallest size of 1 × 1 cm^2^ and the maximum rotational speed of 250 rpm were attained using this configuration. We determined the relationship between the angular velocity of the motor and the input power for different rotor diameters and arrangements of the Lamb wave actuators. The limitation of the device lies in its restricted angular velocity; however, the results provide theoretical guidance for further improvement. This novel motor meets the miniaturization needs and presents new possibilities for silicon-based and integrated microactuation technology. This motor is expected to have applications in point-of-care testing (POCT) devices and other microfluidic systems.

## Data Availability

The data that support the findings of this study are available from the corresponding author upon reasonable request.

## Supplementary Materials

The following supporting information can be downloaded at: https://www.mdpi.com/article/10.3390/mi15060716/s1, Figure S1: The fabrication process of the LWR Arrays; Figure S2: The rotor position at different times.
