# Supplementary material for: Low-Voltage High-Frequency Lamb-Wave-Driven Micromotors
Source: Micromachines (Basel). 2024 May 29;15(6):716. doi: 10.3390/mi15060716 (PMC11206021; doi:10.3390/mi15060716)
Supplement: Supplementary file 1 [file micromachines-15-00716-s001.zip › micromachines-3024856-supplementary.pdf]

1. The fabrication process of the LWR Arrays

The fabrication process (Figure S1) is as follows: starting with a single-side polished, high-resistivity four-inch silicon wafer as the substrate, the procedure initiates by applying the first mask on a clean silicon surface for spin-coating, exposure, and development. Reactive Ion Etching (RIE) is then employed to etch a 3 μm deep cavity into the silicon wafer, serving as the resonant cavity for the Lamb wave resonator. Chemical Vapor Deposition (CVD) is used to deposit phosphosilicate glass (PSG) into the cavity. Sacrificial layers of various silicon oxides (such as silicon dioxide, SiOx, and silicate glass) are commonly utilized in surface microfabrication processes, as they can be selectively etched by hydrofluoric acid (HF) during the subsequent cavity release step, while silicon remains largely unaffected. The height of the PSG should slightly exceed that of the cavity. Chemical Mechanical Polishing (CMP) is subsequently applied to smoothen the cavity edges and substrate interface, ensuring a flat silicon wafer surface for the quality of subsequent film depositions.

Radio Frequency Magnetron Sputtering (RFMS) is then used to deposit a 0.2 μm thick layer of molybdenum (Mo), followed by photolithography steps using the bottom electrode mask and RIE to define the bottom electrode pattern. This is followed by visual inspection and resist removal. In an array of Lamb wave resonators, electrode dimensions significantly influence performance; thus, careful examination of line widths during visual inspection is crucial to verify the etching quality, necessitating a rework including resist removal and re-exposure if standards are not met.

RFMS is further employed to deposit a 1.5 μm thick aluminum nitride (AlN) layer as the piezoelectric layer and another 0.2 μm Mo layer as the top electrode, with the top electrode pattern defined through photolithography and subsequent RIE. Initial RIE is used to partially etch the AlN, followed by a series of light wet etches (using potassium hydroxide solution) to avoid over-etching. The purpose of etching the AlN is twofold: to expose the sacrificial layer for later cavity release and to reveal the bottom electrode for electrical connections. After resist removal, dilute hydrochloric acid is used to cleanse the oxide from the Mo top electrode surface.

Physical Vapor Deposition (PVD) is then implemented to grow a metal gold layer, with the fifth mask utilized for spin-coating, exposure, and development using negative resist. A lift-off technique is employed to obtain the electrode patterns, where gold electrodes serve as electrical connectors between the top and bottom electrodes while also protecting the Mo electrodes from oxidation, thereby prolonging device lifespan. Lastly, the sacrificial PSG layer is etched away using HF, effectively releasing the cavity.

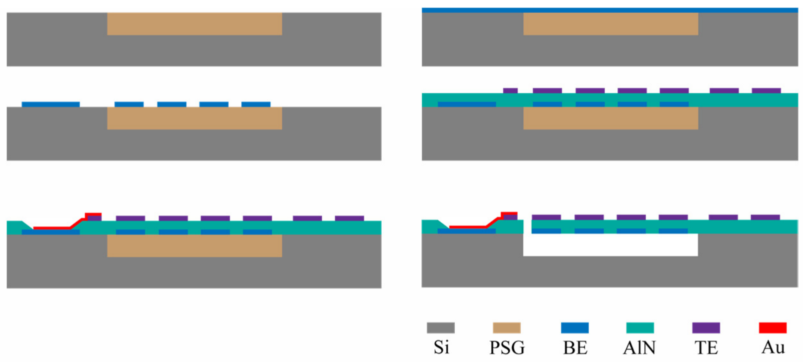

Figure S1. The fabrication process of the LWR Arrays

2. The equipment and reagent manufacturers

The signal generator (N2181B, Agilent) and The power amplifier (QPAR1R53337, Qualwave) and isolator (QCIB-350-430-S, EPool). The high-speed camera is from iSpeed, Olympus (Japan). SU-8 photoresist was purchased from Suzhou KeYi Materials Microtech Co., Ltd. (Suzhou, China).

In general experiment workflow, an excitation signal of a specific frequency which was the resonant frequency of the LWR array measured by vector network analyzer in advance was generated by the signal generator and connected to the device through the power amplifier and isolator. Drop volumes of approximately 50 μL were employed in the central region of the array, and then the motor was carefully placed on the water droplets to maintain a balanced state. As the AC signal was on, the superposition of unidirectional acoustic streaming actuated the rotor to rotate at a certain speed which was measured by a high-speed camera, by the results of which the angular velocities were estimated.

3. The rotor speed measurement

As the AC signal was on, the superposition of unidirectional acoustic streaming actuated the rotor to rotate at a certain speed which was measured by a high-speed camera (iSpeed, Olympus), shown in figure S2. Figure (c) is the superposition of Figure (a) and Figure (b). The fabrication process of the LWR Arrays, Then, we can obtain the angular velocity of rotation by dividing the angle of the two measured photos by the time interval between the two photos. As can be seen, at moments T1 to T2, the rotor rotates $\Delta\omega$. So the rotational speed $S_r = 2\pi\Delta\omega/(T2 - T1)$.

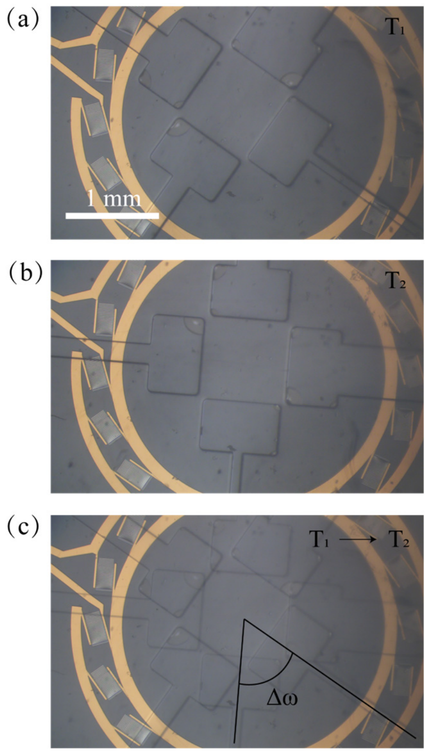

Figure S2. The rotor position at different times

4. Specific steps of COMSOL simulation

The computational workload of the overall model proceeds as follows: Initially, the piezoelectric effect of the Lamb wave resonator and the linear acoustic velocity field within the fluid domain are calculated using the solid mechanics, electrostatics, and pressure acoustics modules to determine the impedance-frequency relationship and the resonant frequency of the resonator. Subsequently, in the fluid domain of the established model, a laminar flow module is applied, accompanied by the introduction of a volumetric force. The velocity field solution is then utilized in the formula for volumetric force, ultimately yielding the simulated acoustic streaming velocity field. For particle simulation, a particle simulation module is further incorporated, where particle trajectories are governed by drag forces from the fluid velocity field and acoustic radiation forces generated by the device.

In the context of a 2D simulation model, the steps are outlined as follows:

A 2D spatial modeling environment is chosen, with the inclusion of "Solid Mechanics," "Electrostatics," and "Pressure Acoustics" modules, each with their respective domains defined. The 2D geometry of the Lamb wave resonator is constructed, comprising a piezoelectric film, electrodes, and the fluid domain. Materials are assigned to the corresponding geometric regions: AlN for the piezoelectric layer, Mo for the electrodes, water for the fluid domain, and Si for the substrate. Loss factors are included, and boundary conditions are set. These encompass radiation boundaries and hard acoustic field boundaries for the fluid domain, along with acoustic-structure boundaries in the multiphysics context. Meshing is performed. Frequency-domain solving is conducted to obtain the displacement field and sound pressure field at the resonant frequency. Through finite element simulation, a 2D model of the novel Lamb wave resonator is established, with resonant frequencies of 380 MHz. In this resonator, the fluid domain near the resonator edges experiences a fluctuating pressure field, and the presence of air gratings on one side channels acoustic wave leakage into the fluid unidirectionally, creating a volumetric force solely on this side. Moreover, the pressure intensity and gradient increase on the air grating side,

enhancing the acoustic streaming effect there.

Owing to the geometric attributes of the resonant cavity, vibrations at its boundaries act as acoustic sources in the liquid and can be conceptualized as "line sources." Consequently, the acoustic waves in the vicinity of the lateral boundaries of the resonant cavity can be interpreted as cylindrical waves. The acoustic streaming phenomenon induced in the liquid by the Lamb wave resonator is precisely triggered by these cylindrical waves. Within the Lamb wave resonator and along its lateral boundaries, the displacement amplitude distribution of its fundamental mode vibration can be mathematically described by Equation S1, where W denotes the electrode stripe width, L signifies the electrode stripe length, and u_0 represents the amplitude of the displacement field.

$$u_s = u_0 cos\left(\frac{\pi}{W}x\right) sin\left(\frac{\pi}{L}y\right) \quad \text{(S1)}$$

Furthermore, in accordance with the wave equation governing cylindrical waves, the velocity fluctuation equation close to the resonator's edge can be formulated as Equation S2, with β representing the attenuation coefficient of the sound wave, k being the wave number, and r symbolizing the propagation distance of the sound wave.

$$\boldsymbol{v}_1 = \frac{\boldsymbol{v}_{10}}{\sqrt{r}} e^{i(-kr+\omega t)} e^{-\beta r} \quad \text{(S2)}$$

Upon extracting the first-order velocity field from the linear acoustic analysis, this information is incorporated into Equation S1 to calculate the volumetric force, which is subsequently introduced into the domain influenced by the volumetric force. The upper boundary of the fluid domain is designated as an "open boundary," effectively simulating an infinitely extended liquid medium. The computational results showcasing the steady-state laminar flow for the innovative Lamb wave resonator are illustrated in Figure 3. Due to the unidirectional characteristic of the volumetric force, the fluid migrates along the y-axis in line with the acoustic wave propagation, eventually circulating back towards the resonator's tail end, thereby generating vortices on both sides (Figure 3(c)).
